# ISMB 2003 Bio-ontologies SIG and Sixth Annual Bio-ontologies Meeting Report

**DOI:** 10.1002/cfg.339

**Published:** 2003-12

**Authors:** Phillip Lord, Robert Stevens

**Affiliations:** Department of Computer Science University of Manchester Oxford Road Manchester M13 9PL UK

## Abstract

The Annual Bio-Ontologies meeting (http://www.cs.man.ac.uk/˜stevens/meeting03/) has now been running for 6 consecutive years, as a special interest group (SIG) of the much larger ISMB conference. It met in Brisbane, Australia, this summer, the first time it was held outside North America or Europe. The bio-ontologies meeting is 1 day long and normally has around 100 attendees. This year there were many fewer, no doubt a result of the distance, global politics and SARS. The meeting consisted of a series of 30 min talks with no formal peer review or publication. Talks ranged in style from fairly formal and complete pieces of work, through works in progress, to the very informal and discursive. Each year's meeting has a theme and this year it was ‘ontologies, and text processing’. There is a tendency for those submitting talks to ignore the theme completely, but this year's theme obviously struck a chord, as half the programme was about ontologies and text analysis (http://www.cs.man.ac.uk/˜stevensr/meeting03/programme.html). Despite the smaller size of the meeting, the programme was particularly strong this year, meaning that the tension between allowing time for the many excellent talks, discussion and questions from the floor was particular keenly felt. A happy problem to have!


**Comparative and Functional Genomics**
*Comp Funct Genom* 2003; **4**: 663–666.
Published online in Wiley InterScience (www.interscience.wiley.com). **DOI:** 10.1002/cfg.339

**Conference Review**

### ISMB 2003 bio-ontologies SIG and sixth annual bio-ontologies meeting report

ISMB'03, Brisbane, Australia, 28 June 2003

Phillip Lord and Robert Stevens*
*Department of Computer Science, University of Manchester, Oxford Road, Manchester, M13 9PL, UK*

*Correspondence to:
Robert Stevens, Department of Computer Science, University of Manchester, Oxford Road, Manchester, M13 9PL, UK.
E-mail:
robert.stevens@cs.man.ac.uk*

Received: 22 September 2003
Revised: 29 September 2003
Accepted: 29 September 2003

### Abstract

**The Annual Bio-Ontologies meeting (http://www.cs.man.ac.uk/~stevens/meeting03/) has now been running for 6 consecutive years, as a special interest group (SIG) of the much larger ISMB conference. It met in Brisbane, Australia, this summer, the first time it was held outside North America or Europe. The bio-ontologies meeting is 1 day long and normally has around 100 attendees. This year there were many fewer, no doubt a result of the distance, global politics and SARS.**

**The meeting consisted of a series of 30 min talks with no formal peer review or publication. Talks ranged in style from fairly formal and complete pieces of work, through works in progress, to the very informal and discursive. Each year's meeting has a theme and this year it was 'ontologies, and text processing'. There is a tendency for those submitting talks to ignore the theme completely, but this year's theme obviously struck a chord, as half the programme was about ontologies and text analysis (http://www.cs.man.ac.uk/~stevensr/meeting03/programme.html). Despite the smaller size of the meeting, the programme was particularly strong this year, meaning that the tension between allowing time for the many excellent talks, discussion and questions from the floor was particular keenly felt. A happy problem to have!**
**Copyright © 2003 John Wiley & Sons, Ltd.**

### Introduction

Biologists long ago passed the point at which more information was produced than one person could read and understand. With the advent of genome data, the need to organize and search has become extreme. The post-genomic era has increasingly meant that biologists wish to make comparisons between species, as well as investigate the biology of a single species. A common understanding of biological knowledge is essential for such comparisons. An ontology is a means of capturing and storing knowledge about a domain, so that it can be shared by both humans and computers. The aim is not to replace the role of the biologist in understanding, describing and explaining biological functions, but to enable them to store, organize and use the knowledge that they, as a community, produce. This in turn allows the more efficient retrieval and searching of biological data. The hope is that a commitment to a common understanding by both humans and computers should enable the scientific process.

There are many ways of expressing and storing artefacts which are broadly covered by the term 'ontology'. These range from an unstructured controlled vocabulary, to a very formal 'description logic' representation of knowledge. The meeting has retreated from arguments about representation and formality to one of pragmatism and biological usefulness.

Although ontologies have been used for many years, the recent explosion of interest within bioinformatics can be explained straightforwardly in three words — 'The Gene Ontology' (GO). The success of GO continued to show at this years meeting, with many talks relating directly or indirectly to it and forming a thread throughout the day.

Copyright © 2003 John Wiley & Sons, Ltd.

Two main themes emerged strongly this year. The first of these was the intended theme of the overlap between text and ontologies, with several speaker's talks about using text techniques to enable GO annotation (**Tony Smith**), as a semantically annotated corpus of free text (**Cliff Joslyn**) or to improve the quality of free text parsing (**Larry Hunter**).

The second theme was the increasing number of bridges being built between the more formal computer science and those more interested in practical applications. Both BioPAX (represented by **Joanne Luciano**), and CellML (represented by **Matt Halstead**), are starting to the use OWL, an ontology language based on an underlying description logic, and stemming from the computer science and semantic web communities. In his second talk, **Cliff Joslyn** discussed applying a more formal mathematical approach to the graph defined by GO, to enable searching and classification of resources annotated with GO. Finally, **Suzi Lewis** talked about the attempts of the sequence ontology (SO) group to build a useful resource and also to use this younger and smaller resource as a test bed for advancing the methodologies for development of SO, which will hopefully feed back into GO. We can only applaud the willingness of many within the bio-ontology community to operate across domains in their attempts to find the right technology fit to provide good solutions to the right problems within bioinformatics.

### Text analysis and bio-ontologies

For many years the main record of biological knowledge has come in the form of text. The scientific journal, or conference paper, is still the most common form of publication. The advent of genomics means that much knowledge is now stored in databases, but much of this is in the form of free text or partially structured data. It is inevitable, then, that bioinformaticians are going to want to extract, analyse, and index this free text. Human understandable languages are, unsurprisingly, good for humans but not so suitable for computers. We need computers to deal with text, even at the level of finding the paper we next wish to read. So improving text analysis has a strong motivation.

Meanwhile, from the computer scientist's perspective biology presents a number of opportunities and interesting challenges. The sheer quantity of text, its unusual use of grammar and vocabulary and the high level of synonymy mean that techniques arising from computer science are tested to the limit.

Ontologies are sometimes seen as an alternative for storing and representing knowledge to free text. Natural language forms of biology's knowledge, however, will always be required for humans; it is more expressive and flexible. Ontologies do, however, give the rather more limited computer some means by which a domain's knowledge become computationally amenable. Text-analysis techniques and ontologies are also sometimes seen as competitors for the same roles — text analysis is necessary where you do not have ontologies, and ontologies are useful so that you do not have to use text analysis. The first session disproved this notion by showing the many ways in which text analysis and ontological technologies can be used to augment each other.

In the first talk, **Tony Smith** discussed linking Medline to GO. Currently the GO annotation database links to many Medline abstracts, but only the minority of Medline abstracts can be linked back to GO terms (110 000 out of 6 000 000). This talk discussed the experiences of applying machine learning techniques to derive GO annotations for all Medline abstracts. Interestingly, the large size of Medline and GO makes many machine learning techniques unfeasible. It appears that the hard work of the annotators has presented a difficult computational challenge for the computer scientists. Using their technique, the human-curated training sets are only large enough to model about one quarter of GO terms, suggesting that in this case computer science can present some interesting challenges to the annotators. The results of this analysis are available at **http://www.go-kds.com**

From the perspective of the ontologist, the information in GO is encoded in the terms and the relationships. However, there is also a large amount of information in the noun phrases that form the terms, e.g. glucose metabolism. In his first talk, **Cliff Joslyn** used natural language techniques to extract lexical data from these terms. This has the advantage that this lexical data is associated with the GO terms, which provide a specification of their meaning, e.g. many GO terms, such as 'lipoprotein

Copyright © 2003 John Wiley & Sons, Ltd. 

metabolism', or 'protein biosynthesis' are of the form 'entity process'. The two main advantages of this are that, first, GO can become a source of lexical data, which can be used to enable mining of non-GO text resources, and secondly, text analysis can be used to identify parts of GO where terms have been used inconsistently or incorrectly.

In related work, **Larry Hunter** described the use of ontologies to drive text analysis. In particular, he showed how the knowledge represented in an ontology can be used to help disambiguate words with several meanings, which is a recurrent problem in bioinformatics, e.g. the noun 'hunk' has several meanings within bioinformatics (and several more in common use!) 'Hunk'; the gene often occurs in sentences with verbs such as 'expressed', or 'regulated'. The combination of text analysis and GO was able to disambiguate between the use of Hunk to describe the gene, as opposed to other entities with the same name.

In this work, GO was used as the primary knowledge resource. This was used to extract disease–gene associations from Medline. Once again, the usefulness of GO as a resource for text extraction is clear.

This section of the meeting was concluded with a talk from **Matthew Wright**, from the HUGO Gene nomenclature committee. Gene naming is not strictly ontological, but rather terminological. However, the main reason for providing these standards is to generate a source of common understanding. It is also clear that for many of those carrying out text analysis, fewer synonyms in biology would be a welcome relief.

HUGO (**http://www.gene.ucl.ac.uk/nomenclature/**) has now provided names for half of the human genes. Their procedure involves a rigorous analysis of each gene to determine that it is different from all others, including those which are not publicly available. This talk was followed by a particularly lively discussion of HUGO's use of a simple name to try and convey complex information about complex concepts, such as a protein, its function and familial relationships.

### Ontologies: news and views

Following the usual very loose approach to the theme during bio-ontologies meetings, the second half of the day was given over to 'news and views'; presentations about new ontology projects, the application of ontological technology outside of text analysis, and updates on existing ontology projects. These three areas have been recurrent themes throughout the life of the bio-ontologies meetings. It is pleasing to see that the breadth of talks has spread over the years.

There has been a long history of the use of ontologies to enable accurate and expressive information retrieval. The ontology can be used to index database records, documents or other entities. To work well, the ontology needs to cover large parts of the domain. In the first talk of this section by **Nick Tilford** of BioWisdom (**http://www.biowisdom.com**), we heard about a number of different approaches to generating these large-scale ontologies and their use within the drug discovery process.

Although biologists have been interested in pathways for many years, genome technologies have resulted in the development of many pathway databases. The need for a common mechanism for accessing and transferring this data is pressing. In her talk, **Joanne Luciano** described the current efforts of the BioPAX working group (**http://www.biopax.org**) toward providing such a mechanism. The group is going to great efforts to address the user requirements of the community, and is working in collaboration with the authors of other mark-up languages, such as SBML and CellML, which address related domains. They are also investigating the use of both XML schema and OWL (the Ontology Web Language) as mechanisms for the BioPAX format.

Text processing in bioinformatics largely revolves around the application of statistical techniques to the words produced by biologists. In his first talk, **Cliff Joslyn** had discussed applying these techniques to the terms in GO. In his second talk he discussed applying similar techniques to the structural graph produced by the relationships between the terms. A number of different methods were shown for clustering terms with GO. These techniques are interesting as they may provide new applications for GO, particularly for experiments such as microarrays, which produce large numbers of proteins or genes that need analysing and organizing.

The sequence ontology (**http://song.sourceforge.net**) is one of the new ontologies coming out of the 'open biological ontologies' initiative.

Copyright © 2003 John Wiley & Sons, Ltd.

(**http://obo.sourceforge.net**) Its aim is to describe features of sequences, such as mutation sites or TATA boxes. It is already coming into use as part of GFF, the file format for transferring sequence feature information.

We were expecting a talk on an important project in its early stages. **Suzi Lewis**, however, treated us to a discursive and lively talk largely relating to the difficulties in understanding 'part-of' relationships within bioinformatics. This discussion has a long history within the ontology community, where a distinction is often made between statements such as 'the morning is part of the day', and 'a finger is part of a hand'. It will be interesting to see how much of this debate can usefully feed into the bio-ontologies community.

**Matt Halstead's** talk on the Physiome Project described an attempt to build a comprehensive framework for computational modelling of human biochemistry, biophysics and anatomy. The goal of this project is to use computational modelling to analyse integrative physiological function in terms of underlying biological structure and processes. The framework includes several databases that describe organs, tissues and processes at many levels. These resources hold quantitative, mathematical and bibliographic data about cellular processes. All these data are organized within CellML(**http://www.cellml.org**) and CellML Metadata XML representations. All these data and schemata carry implicit and unconnected data about systems biology. The Physiome Project has been working on an OWL ontology to capture this knowledge and make it explicit within the models, helping to avoid semantic errors within the models, to support and guide both user and programme through complex processes.

It is clearly of interest to be able to describe disease, and disease features, in the hope that the predications of 'genotype to phenotype' can start to come into reality. Of course, describing diseases ontologically is not a new idea, with classifications such as ICD 9 having a long history. **Patricia Dyck's** talk, however, highlighted a number of problems with existing ontologies, e.g. that most are not freely available, and that they are largely written from a medical, rather than a biological, perspective. The disease ontology (**http://diseaseontology.sourceforge.net**) has already reached a relatively large size (over 6000 terms) and is attempting to address these issues, in particular promoting the biological viewpoint of disease.

In the final talk of the day, we were told about the application of the Gene Ontology to the Rat Genome Database (**http://rgd.mcw.edu/**). In this case, a careful software development process was undertaken to ensure that all the requirements of the many users of this database were fulfilled. The results of this are many new features, in particular search facilities, driven by GO, which we hope will prove popular in use.

### Summary

As each year goes by bio-ontologies are moving toward being an accepted technology within the bioinformatics area. Each year the bio-ontology meeting becomes stronger and stronger. To a large extent the community has left behind sterile arguments like 'What is an ontology?' and is keen to pursue questions such as 'How do we use ontologies to enable us to do better biology?', and 'How can we make use of biological data to drive forward improvements to ontological methodologies?'. Computer scientists should see biology as a domain to test their technologies and methodologies. By so doing and showing that their views provide greater utility, the differing agenda of the two communities can coexist. With this in mind, we look forward to next year's meeting in Glasgow.

### Acknowledgements

Phillip Lord is supported by the myGrid e-Science pilot project grant (EPSRC GR/R67743).

Copyright © 2003 John Wiley & Sons, Ltd. 

